# *Smart Secondary Metabolites* in Marine Environments: Exploring the Oxasqualenoid Dehydrothyrsiferol

**DOI:** 10.3390/md24050155

**Published:** 2026-04-27

**Authors:** Francisco Cen-Pacheco, Ana R. Díaz-Marrero, José J. Fernández

**Affiliations:** 1 Facultad de Bioanálisis, Campus-Veracruz, Universidad Veracruzana, Veracruz 91700, Mexico; 2 Instituto de Productos Naturales y Agrobiología (IPNA), Consejo Superior de Investigaciones Científicas (CSIC), Avenida Astrofísico Francisco Sánchez 3, 38206 La Laguna, Spain; 3 Biotecnología Marina, IUBO-ULL, Unidad Asociada al IPNA-CSIC, 38206 La Laguna, Spain; 4 Instituto Universitario de Bio-Orgánica Antonio González (IUBO AG), Universidad de La Laguna (ULL), Avenida Astrofísico Francisco Sánchez 2, 38206 La Laguna, Spain; 5 Departamento de Química Orgánica, Universidad de La Laguna (ULL), Avenida Astrofísico Francisco Sánchez 3, 38206 La Laguna, Spain

**Keywords:** *Smart Secondary Metabolites*, dehydrothyrsiferol, *Laurencia viridis*, oxasqualenoids, drug discovery

## Abstract

Dehydrothyrsiferol (DT), a brominated oxasqualenoid from the red alga *Laurencia viridis*, represents a compelling example of this framework. This review establishes DT as a model *Smart Secondary Metabolite* based on the convergence of a unique molecular architecture of rigid stereogroups connected by flexible bonds; a high metabolic yield (0.42% *w*/*w* of crude extract); potent selective bioactivity against kinetoplastids and drug-resistant tumors; multi-target modulation of protein phosphatase 2A (PP2A) and cell-surface integrins; and distinctive chemotaxonomic relevance within Macaronesian communities. Its biosynthesis proceeds through stereocontrolled epoxide-opening cascades, generating an evolutionarily refined scaffold. Ecologically, DT operates as a multifunctional shield, providing antifouling protection and deterring herbivory. Pharmacologically, it acts as a selective signaling modulator, triggering integrin-mediated cell death (IMD) in resistant cancer cells and inducing mitochondrial collapse in protozoa. In vivo studies in murine models of cutaneous leishmaniasis have demonstrated an 87% reduction in lesion size, reinforcing its promise as a lead structure.

## 1. Introduction

The marine environment has long been recognized as a prolific source of natural products, traditionally used by coastal cultures for nutrition and medicinal purposes [1]. Among marine organisms, seaweeds possess an exceptionally sophisticated chemical machinery that has evolved under intense ecological pressures. This evolutionary context has resulted in the production of structurally diverse and biologically potent metabolites whose features extend far beyond those typically achieved through synthetic chemistry [2,3,4]. Within this complex biochemical landscape emerges a particularly significant category of compounds that can be conceptualized as *Smart Secondary Metabolites* (*SSMs*), a class of natural products that embodies the most functionally advanced outcomes of marine chemical evolution. These metabolites do not merely occur as by-products of metabolism; rather, they represent integrated biochemical solutions shaped by millions of years of ecological optimization [5].

*SSMs* are distinguished by the convergence of several defining attributes:

- *Singular chemical architectures*, often stereochemically dense and structurally unique, reflecting highly specialized biosynthetic pathways.
- *High metabolic productivity*, despite their non-essential role in basal cellular functions, suggesting adaptive value and potential roles as precursors for related molecular families.
- *Potent and multifaceted biological activities*, including antimicrobial, anticancer, anti-inflammatory, and antiparasitic effects.
- *Intrinsic multi-target interaction capacity*, enabling them to modulate different molecular pathways, either through selective binding or controlled promiscuity.
- *Ecological and chemotaxonomic significance*, reflecting their importance in species survival strategies and their usefulness for phylogenetic differentiation.

When viewed through this framework, marine natural products (MNPs) can be understood as an expansive reservoir of ecologically validated *SSMs*. These compounds are not random or incidental; instead, they are the biochemical products of strong abiotic and biotic selection forces that have consistently favored molecular designs capable of interacting with conserved biological targets with exceptional specificity and affinity [6,7,8].

This theoretical lens shifts the paradigm of drug discovery. Rather than screening vast synthetic libraries in search of meaningful activity, researchers can prioritize metabolites whose ecological behavior already predicts their translational potential. In this sense, ecosystems act as pre-laboratory screening platforms, refining chemical scaffolds over evolutionary time [9]. For instance, antifouling metabolites that prevent bacterial adhesion reflect mechanisms relevant to inhibiting cancer cell metastasis [10]; allelopathic agents that disrupt competitors’ metabolic pathways often expose mitochondrial vulnerabilities in pathogenic eukaryotes [11]; ichthyotoxic defenses have historically guided the elucidation of neuroactive ion-channel modulators [12]; quorum-sensing compounds suggest innovative approaches to attenuate antimicrobial resistance by disabling pathogenic coordination rather than killing the organisms outright [13].

Thus, incorporating the concept of *SSMs* into marine bioprospecting frameworks provides not only a classification tool but also a predictive strategy for identifying compounds with the highest biomedical promise. By understanding these metabolites as the optimized chemical probes of marine ecosystems, researchers can accelerate the development of novel treatments for antimicrobial-resistant infections, neglected tropical diseases, and highly refractory cancers [5].

**Figure 1:**
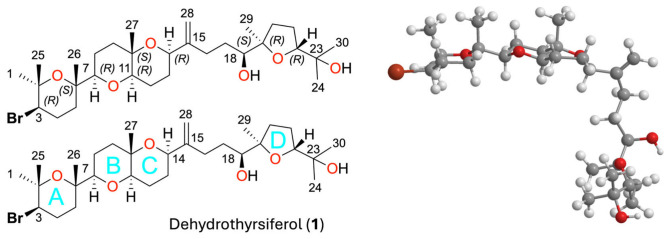
Molecular architecture of dehydrothyrsiferol (DT). The skeletal structure illustrates the sophisticated design of this major marine oxasqualenoid, characterized by an alternating ‘rigid-flexible’ framework. This specific architectural arrangement provides the necessary spatial adaptability for the molecule to adopt its characteristic ‘hook-like’ or ‘L-shaped’ global conformation, establishing DT as a highly versatile multi-target metabolic chassis. Numbering system indicates atom position, *R* and *S* configuration of stereocenters and rings A–D have been identified, oxygen atoms indicated in red.

Within this vast biological reservoir, marine macroalgae emerge as a key source of bioactive molecules, characterized by unique metabolic profiles and sophisticated biosynthetic machinery [14]. Among them, the genus *Laurencia* (Ceramiales, Rhodomelaceae) is recognized as one of the most prolific and chemically diverse groups of marine organisms. Renowned for its exceptional biosynthetic capacity to produce complex halogenated metabolites, this genus has been at the forefront of marine natural products research for decades [15,16,17,18]. Within this vast chemical repertoire, triterpene polyethers (oxasqualenoids) represent one of the most sophisticated classes of secondary metabolites, with dehydrothyrsiferol (DT, Figure 1) emerging as a hallmark of structural complexity and biological potency [19].

Endemic to the Macaronesian region, *L. viridis* forms a distinctive component of benthic communities in the Canary Islands, where it occupies the lower intertidal zone exposed to intense hydrodynamic stress [20,21,22]. Unlike the perennial growth patterns of many congeneric species, *L. viridis* exhibits a strict annual cycle: thalli proliferate rapidly in winter and spring, reach peak biomass in early summer, and senesce shortly thereafter. This temporal rhythm is tightly synchronized with its chemical output [21,22,23,24]. During its short life span, the species undergoes a dramatic metabolic investment in producing DT, suggesting strong evolutionary pressure favoring this single metabolite as the species’ primary biochemical defense [25,26].

Chemically, *L. viridis* constitutes an anomaly within the *Laurencia* genus. While most species generate a broad spectrum of halogenated sesquiterpenes and acetogenins [15,27,28], *L. viridis* diverts its biosynthetic flux almost entirely toward polyether triterpenes, with a dominant production of DT [23,29]. The high prevalence of DT highlights an evolutionary commitment to a single, structurally extreme metabolite whose molecular architecture exceeds the stereochemical density and functional complexity typical of marine natural products [19,30].

DT’s architecture, built upon a squalene-derived polyether skeleton containing multiple cyclic tetrahydropyran units, oxygen-rich linkages, and densely packed quaternary stereocenters, positions it at the apex of marine terpenoid complexity. Its rigid stereoclusters, balanced by flexible chains, create a dynamic yet highly specific conformational landscape that enables selective engagement with biological macromolecules [19].

## 2. Dehydrothyrsiferol as a Canonical Smart Secondary Metabolite

The biochemical and ecological characteristics of DT exemplify the defining criteria of an *SSM*, making it an exemplary model of this conceptual category [5]:

- *Singular molecular architecture*: The molecule exhibits extreme stereochemical richness, modular cyclization, and oxygen-dense polyether domains that enable precise molecular recognition. This architecture provides the necessary spatial adaptability for the molecule to adopt its characteristic ‘hook-like’ or ‘L-shaped’ global conformation, functioning as a versatile multi-target metabolic chassis [19].
- *High metabolic yield*: *L. viridis* allocates an extraordinary proportion of its metabolic resources to DT biosynthesis, elevating it from a secondary product to a dominant biochemical signature of the species [29].
- *Potent biological activity*: DT displays nanomolar or low micromolar potency across multiple eukaryotic systems, reflecting strong target engagement. From the biomedical point of view, DT exhibits an exceptional degree of biochemical precision, positioning it as a strategic inhibitor of key eukaryotic processes. Notably, DT acts as a selective inhibitor of protein phosphatase 2A (PP2A) [31], a central regulator of cell proliferation and signaling pathways, placing it among the most structurally intricate natural modulators of this enzyme. In addition, DT modulates the affinity of VLA integrins [32], thereby influencing adhesion-dependent signaling with remarkable specificity. Equally significant is its ability to evade multidrug resistance mechanisms: DT remains highly active in cancer cell lines overexpressing P-glycoprotein efflux pumps [33], retaining its potency even in phenotypes that typically undermine standard chemotherapeutic agents.
- *Multi-target capability*: Though capable of modulating several molecular pathways, its activity remains highly specific to defined regulatory nodes, illustrating controlled, evolutionarily optimized ‘promiscuity’. These properties not only demonstrate multi-target capacity but also reveal an inherent selective hierarchy. DT engages multiple pathways, but only those that share conserved structural or regulatory features across eukaryotes. The remarkable breadth of DT’s bioactivity, from human cancer cells [34,35,36] to protozoan pathogens such as *Leishmania* [37] and *Acanthamoeba* [38], reflects a molecule honed to disrupt universal eukaryotic vulnerabilities.
- *Chemotaxonomic value*: The predominance of DT is so characteristic of *L. viridis* that its presence is effectively diagnostic of the species.
- *Ecological intelligence of DT in its native environment*: In the intertidal ecosystem, DT functions primarily as a highly specialized antifouling agent. Its capacity to inhibit the settlement and growth of benthic diatoms and macroalgal propagules (e.g., *Gayralia oxysperma*) reflects a fine-tuned ecological adaptation that protects *L. viridis* during vulnerable phases of its annual cycle [25]. The deployment of such a potent metabolite highlights the ecological intelligence embodied by DT, an evolutionary solution optimized to secure survival in a high-competition, high-disturbance habitat.

This cross-system potency reinforces DT as a paradigmatic *SSM*: a natural product shaped by ecological constraints into a precision tool with translational potential.

## 3. Search Strategy for Studies on Dehydrothyrsiferol

This systematic review follows the PRISMA 2020 guidelines (Preferred Reporting Items for Systematic Reviews and Meta-Analyses) [39] to deliver a comprehensive descriptive analysis of the existing literature on dehydrothyrsiferol (DT). A broad bibliographic search was executed across four primary databases: Web of Science (WoS), ScienceDirect, SciFinder-n, and PubMed, spanning the period from the first isolation of the compound in 1984 through February 2026.

The search protocol employed exact phrase queries using the terms ‘dehydrothyrsiferol’, ‘*Laurencia viridis*’, and ‘triterpene polyethers’ across all available fields. These keywords were applied as literal strings without the use of Boolean operators (AND, OR) for combination. The eligibility criteria were strictly defined to include peer-reviewed journal articles published in English that investigated DT from natural sources within taxonomical, ecological, biosynthetic, structural, or pharmacological frameworks.

To guarantee data consistency, the selection was limited to original research articles. Consequently, other publication formats such as conference abstracts, books, book chapters, editorials, and technical reports were excluded. Additionally, patent databases (Lens.org and Espacenet) were consulted without temporary restrictions to assess industrial applications. A detailed breakdown of the search methodology and selection workflow is provided in the Supplementary Materials.

The screening process yielded a total of 257 records: WoS (*n* = 47), ScienceDirect (*n* = 97), SciFinder-n (*n* = 77), and PubMed (*n* = 36). Following the removal of 114 duplicate entries and the exclusion of 63 records based on title and abstract screening, 80 articles were assessed for full-text eligibility. Of these, 42 were discarded for failing to meet the inclusion criteria. Finally, 38 studies were selected for this review (Figure S1).

## 4. Biogenesis of Dehydrothyrsiferol

### 4.1. Biosynthetic Pathway for Dehydrothyrsiferol

Red algal terpene biosynthesis can be divided into four main sequential stages: the biosynthesis of isoprenoid precursor molecules, the linear condensation of these precursors to generate polyisoprenyl diphosphate intermediates, the terpene synthase–catalyzed conversion of these intermediates into core terpene structures, and the subsequent enzymatic modifications that further diversify the basic terpene carbon skeleton [40].

The first stage of terpene biosynthesis generally begins with the linear condensation of two five-carbon isoprenoid precursors: isopentenyl diphosphate (IPP) and its allylic isomer, dimethylallyl diphosphate (DMAPP). These essential building blocks are produced through two distinct metabolic pathways: the mevalonate (MVA) pathway and the 2-C-methyl-D-erythritol 4-phosphate (MEP) pathway [41]. Over evolutionary time, different groups of organisms have developed the ability to generate these terpene precursors by specializing in one or, in some cases, both of these biosynthetic routes. Both biosynthetic routes have been described for *Laurencia* spp. [40].

Dehydrothyrsiferol is an oxasqualenoid that is biosynthetically derived from squalene, a triterpenic precursor common to this family of metabolites. The synthesis of squalene in *Laurencia* is carried out through the mevalonate (MVA) pathway, whose presence has been experimentally demonstrated in *L. dendroidea* via transcriptomic and cytological analyses. Key genes of this metabolic route have been identified as responsible for generating the isoprenoid precursors IPP and DMAPP from acetyl-CoA. These precursors are subsequently elongated by prenyltransferases, also detected in *Laurencia*, to produce geranyl pyrophosphate (GPP) and finally farnesyl pyrophosphate (FPP), the direct precursor of squalene. Next, two molecules of FPP are combined in a reductive dimerization reaction catalyzed by squalene synthase (SQS), an enzyme whose involvement is inferred from the identification of all necessary terpenoid-biosynthesis genes in the *Laurencia* transcriptome, thereby completing the formation of squalene, which serves as the starting point for the diverse and highly halogenated terpenes characteristic of this genus [41].

**Figure 2:**
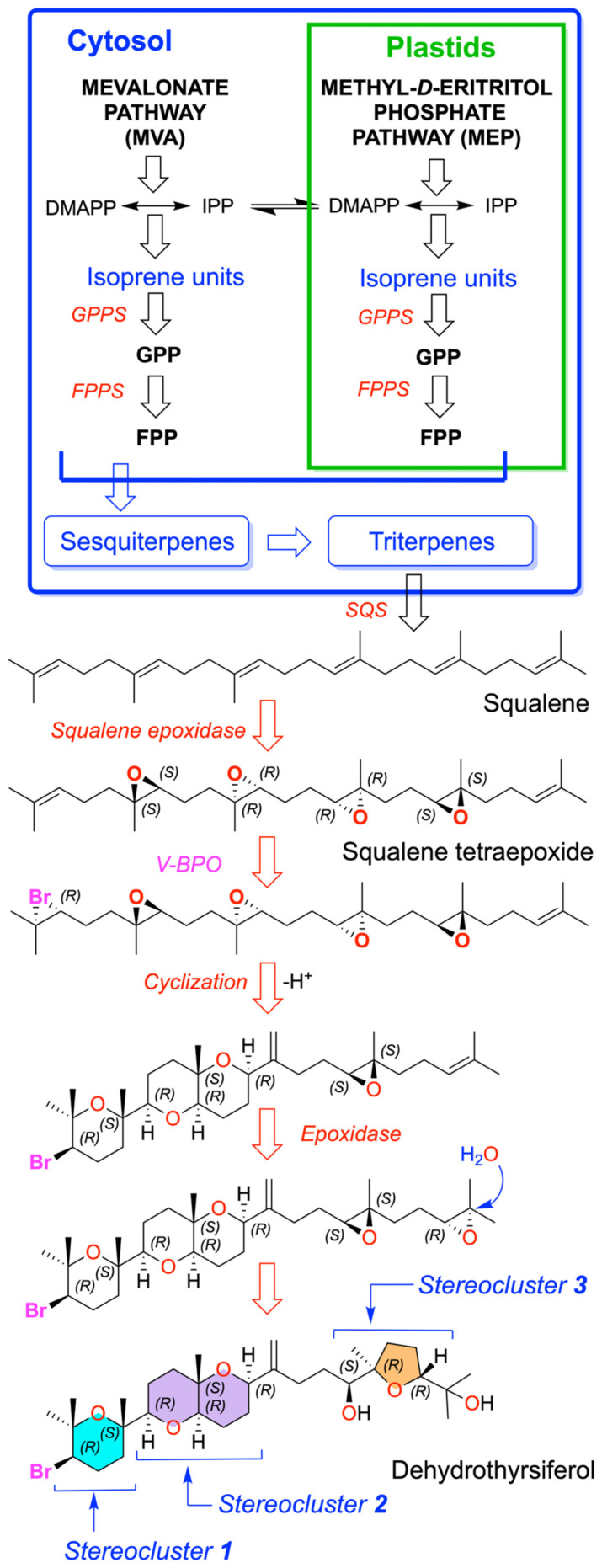
Biosynthetic pathway for dehydrothyrsiferol.

From that point, its formation would involve an initial stage of squalene polyepoxidation, probably mediated by squalene epoxidases to generate a tetraepoxide intermediate [19]. This intermediate would possibly undergo a bromination step typical of the halogenated metabolites of *Laurencia*, presumably mediated by vanadium-dependent haloperoxidases [42], followed by a complex sequence of cascade cyclizations that generate the polyether ring system characteristic of the thyrsiferol family, whose ABC core (stereoclusters 1 and 2) is shared with congeners such as isodehydrothyrsiferol and has been confirmed through total synthesis [43]. Cyclization of the ABC system would lead to the formation of a carbocation at C-15, which would yield dehydrothyrsiferol via deprotonation or thyrsiferol through water addition. Finally, the terminal fragment that produces the D ring (stereocluster 3) can be rationalized by a water-induced cyclization of a diepoxide, as proposed in Figure 2. Overall, although the structure and triterpenic origin are firmly established, the enzymes and specific steps of dehydrothyrsiferol biosynthesis remain unidentified and can only be inferred from general biogenetic models applied to its chemical family.

### 4.2. Metabolic Storage and Transport: The Role of ‘Corps en Cerise’

**Figure 3:**
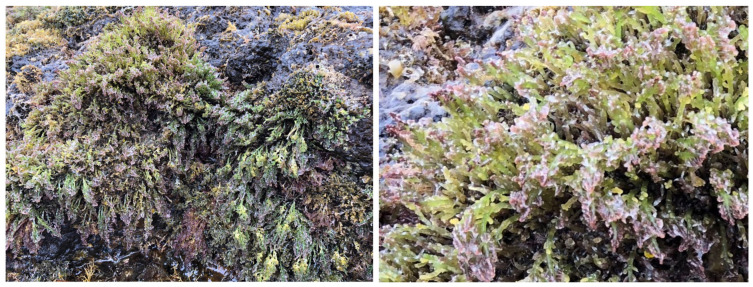
Ecosystem of *Laurencia viridis* in the intertidal zone, Canary Islands.

The production of halogenated compounds such as DT in *L. viridis* (Figure 3) is spatially confined to specialized intracellular organelles known as corps en cerise (CC). First identified via X-ray microanalysis in *L. snyderae* [44], these spherical, electron-dense inclusions are characteristic of the *Laurencia* complex [45]. These organelles function not merely as storage depots of DT, but as active biosynthetic loci that sequester the highly reactive halogenated terpenes, thereby preventing endogenous toxicity to the algal cytoplasmic machinery [44,46].

The mobilization of these defenses involves a highly sophisticated trafficking system. It has been demonstrated that the CC are not static; rather, metabolite-loaded vesicles are actively transported from the CC to the cell periphery via the cytoskeleton, specifically interacting with actin microfilaments [47]. Upon reaching the plasma membrane, these vesicles align with microscopic channels connecting to the cell wall, facilitating the exocytosis of their toxic cargo [46].

Most recently, genomic and biochemical analyses have implicated ATP-binding cassette (ABC) transporter proteins in this excretory process [48]. These transmembrane pumps likely regulate the efflux of hydrophobic oxasqualenoids like DT against concentration gradients, effectively creating a ‘chemical boundary layer’ on the thallus surface that deters fouling organisms and herbivores [46,48]. The high metabolic yield of DT in *L. viridis* [29] necessitates such a specialized transport network; this system ensures that these *SSMs* are precisely deployed at the algal-water interface, exactly where critical ecological interactions occur.

Within this framework of enzymatic precision and energetic commitment, the production of DT in *L. viridis* represents a sophisticated paradigm of metabolic economy. Although overcoming the stereoelectronic constraints of Baldwin’s rules through specialized microenvironments [49,50,51] imposes a significant ‘metabolic tax’, the organism offsets this cost through extraordinary regioselectivity. Far from yielding inactive by-products, the biosynthetic machinery is so refined that the resulting pool of secondary oxasqualenoids constitutes a functional chemical arsenal, providing a multi-layered defense system tailored for diverse ecological interactions [52,53,54].

### 4.3. Structural Features of Dehydrothyrsiferol

Dehydrothyrsiferol (DT, C_30_H_51_BrO_6_) represents a structural paradigm within the oxasqualenoid family [19,24]. Its architecture is defined by three rigid stereoclusters interconnected by flexible acyclic segments, a design that balances structural integrity with the conformational adaptability required for biological binding [26,29]. Thus, stereocluster 1 consists of a brominated tetrahydropyran (ring A) harboring two chiral centers (3R, 6S). Stereocluster 2 features the typical trans-fused dioxabicyclodecane system (rings B–C), providing a robust central core with 7R, 10S, 11R, 14R configurations. Finally, stereocluster 3 is composed of a substituted tetrahydrofuran (ring D) with 19R and 22R centers. These clusters are interconnected by flexible acyclic linkers, notably the C15–C18 bridge containing the 18S stereocenter, which is arguably the most critical region for its pharmacophoric expression [19,25]. Notably, the isolation of DT as the major secondary metabolite (0.42% *w*/*w* relative to the crude extract and 0.005% of dry weight of *L. viridis*) ensures its role as the primary structural template with extensive multi-target profiling [29]. A Monte Carlo (MM2) conformational search with NOESY distance constraints and a 50 kJ/mol energy cutoff identified stable structures which, supported by NMR chemical shift correlations with crystallographic models of thyrsiferyl 23-acetate [55] and venustatriol [56], suggest that DT adopts a non-linear, but rather a ‘hook-like’ or ‘L-shaped’ global conformation in solution (Figure 1) [29].

### 4.4. NMR Strategies for Structural Elucidation

The structural determination of Marine Natural Products (MNPs) is primarily performed using Nuclear Magnetic Resonance (NMR) methodologies [30]. For DT and related oxasqualenoids, these experiments have transcended routine characterization, serving as pivotal models for implementing and validating advanced structural methodologies. The structural “bottleneck” posed by the density of quaternary carbons and flexible acyclic segments in the DT scaffold has promoted the combined use of various spectroscopic strategies with computational or semisynthetic methods [26,53,57].

The relative stereochemistry of the three rigid stereoclusters (rings A, B–C, and D) in DT was independently established through exhaustive dipolar correlations (NOE and ROESY) within each system [25,26]. To overcome the structural ‘bottleneck’ posed by the flexible linkers, the connectivity between the stereoclusters to define the global relative configuration was achieved through chemical correlation. Specifically, the hydration of the C15(28) exocyclic double bond of DT yielded a product whose spectroscopic data (^1^H and ^13^C NMR) confirmed that DT follows the stereochemical pattern of thyrsiferol [25,58]. This distinction is critical, as the thyrsiferol series differs from the venustatriol series specifically in the configuration of the C18 and C19 stereocenters [25,29,56]. Furthermore, this assignment is consistent with a common biogenetic origin from a squalene-derived precursor, providing a robust framework for the proposed relative stereostructure [19,59].

The structural reliability of the rigid stereoclusters in DT has been further corroborated through the indirect application of Density Functional Theory (DFT) on its structural analogues. Specifically, the configurations of ring A and the rings B–C framework were validated in metabolites such as laurokanol A [53] and 15,16-epoxythyrsiferols A and B [26], which exhibit ^13^C NMR chemical shifts for these moieties that are similar to those of DT. In the same way, the structural integrity of ring D is supported by the identical ^13^C NMR shifts observed for the C19–C23 segment in 15,16-epoxythyrsiferols [26]. In these studies, Boltzmann-weighted magnetic shielding constants were computed at the B3LYP/LAVCP**+ or B3LYP/6-31G(d,p) levels of theory. The high correlation between experimental and theoretical data, statistically reinforced by CP3 and *J*-DP4 parameters (probabilities > 99.9%), provides a modern QM-based validation of the tricyclic core in DT. This combined strategy ensures the configurational consistency of the entire oxasqualenoid skeleton, bridging the gap between classical chemical correlation and modern computational validation.

### 4.5. Biogeographic Context: From Global Rarity to Local Dominance

While sesquiterpenes such as elatol are ubiquitous within the ‘*Laurencia* complex’ throughout tropical and temperate oceans [5], triterpene polyethers represent a considerably more restricted biogeographic phenomenon. Historically, the first member of this class, thyrsiferol, was isolated in 1978 from *L. thyrsifera* in New Zealand [58], establishing the South Pacific as the initial *locus* of discovery. This southern distribution was significantly reinforced by the isolation of aplysiols C–E from *Chondria armata* collected at Orpheus Island on the Great Barrier Reef, Australia [60]. This finding is chemically and taxonomically pivotal, as it confirmed that the complex enzymatic machinery required for squalene polyepoxidation is not restricted to *Laurencia* but is also expressed in the closely related genus *Chondria*. Moving northward to the Western Pacific, Japanese waters have proven to be an important reservoir for these metabolites. Distinct structural classes were elucidated across the Japanese archipelago, ranging from the teurilene and magireol series in *L. obtusa* at Teuri Island [55,61], to venustatriol in *L*. *venusta* from Okinawa [56]. This distribution extends to the central Pacific coast of Japan, where enshuol was isolated from *L*. *omaezakiana* [62], and further south to the Gulf of Thailand, with the discovery of callicladol in *L*. *calliclada* from Vietnam [63]. Additionally, in the South China Sea, laurenmariannol and (21*α*)21-hydroxythyrsiferol were isolated from *L*. *mariannensis* [64].

The ecological persistence of these scaffolds is further evidenced by their bioaccumulation in specialized opisthobranch grazers. For instance, *Dolabella auricularia* in Japan and *Aplysia dactylomela* in the South China Sea and off the southwest coast of Puerto Rico have been shown to sequester compounds such as aurilol, aplysiols A–B, and aplysqualenol A and B, respectively [65,66,67], confirming the trophic transfer of these compounds within benthic communities.

**Figure 4:**
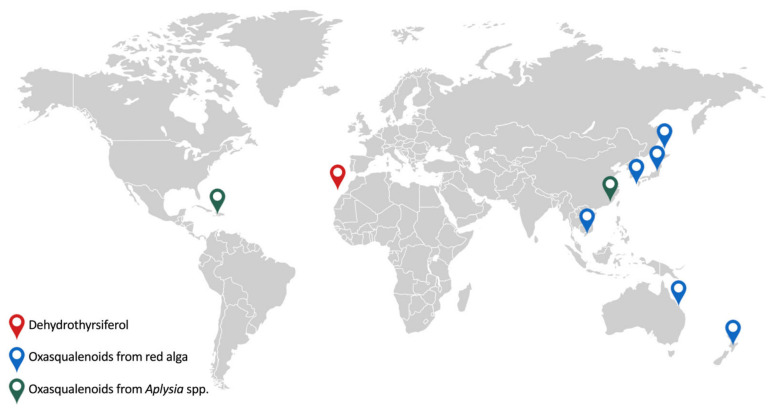
Global biogeographical distribution and chemotypic diversity of representative oxasqualenoids. The map illustrates the localized production of specific polyether scaffolds across the oceans. Crucially, it highlights the Canary Islands (Macaronesian region) as the global biogenetic epicenter, where the endemic *Laurencia viridis* acts as an unparalleled chemical factory producing over 70 distinct oxasqualenoid derivatives, with dehydrothyrsiferol (DT) as the major biosynthetic chassis. Furthermore, the geographical mapping tracks the trophic transfer and metabolic upgrading of these algal precursors by the specialist sea hare *Aplysia dactylomela*, resulting in region-specific defensive derivatives such as aplysiol B (South China Sea) and aplysqualenol A (Caribbean Sea).

However, these global reports typically describe the isolation of single metabolites or small clusters within a species. In stark contrast, the Macaronesian endemism *L. viridis* (originally identified as *L*. *pinnatifida* in Tenerife) represents a biosynthetic singularity. Since the first isolation of dehydrothyrsiferol in 1984 [25], this species has been shown to produce an unprecedented library of over 70 distinct polyethers, marking the Canary Islands not merely as another location, but as the absolute global epicenter of oxasqualenoid chemodiversity (Figure 4).

## 5. Ecological Roles

From an ecological perspective, the biogenetic pathway reveals a deliberate metabolic funneling in *L. viridis*. While thyrsiferol serves as the direct oxygenated precursor, it is isolated only in trace amounts compared to its dehydrated derivative, DT, which accumulates massively (up to 0.42% *w*/*w*) [29]. This overwhelming preference for the dehydrated form suggests that the terminal enzymatic dehydration is not a random artifact, but a highly selected evolutionary refinement. Rather than representing the ultimate, maximally toxic end-product, DT may function as a ‘quasi-ready’ metabolic chassis. By predominantly storing the dehydrated scaffold (DT) within the CC, *L. viridis* likely maintains a baseline chemical defense that is sufficiently stable and manageable, thereby minimizing potential autotoxicity to its own cellular machinery [68]. Under this hypothesis, DT acts as a biochemical weapon ‘with a safety lock’: a poised architectural chassis that, upon exudation or immediate environmental stress, could undergo minor structural modifications to be drastically potentiated into highly toxic derivatives. This strategy would represent a remarkable evolutionary compromise—ensuring safe, high-volume intracellular storage while retaining an adaptable arsenal ready for immediate, targeted activation.

### 5.1. Antifouling Activity and Surface Defense

Sessile marine organisms are subject to constant colonization by microorganisms and invertebrates, a process known as biofouling that can impair photosynthesis and increase hydrodynamic drag [69,70]. To counteract this, macroalgae often employ chemical defenses to maintain a clean thallus surface. In the case of *L. viridis*, the oxasqualenoid dehydrothyrsiferol (DT) appears to play a central role in this strategy.

**Figure 5:**
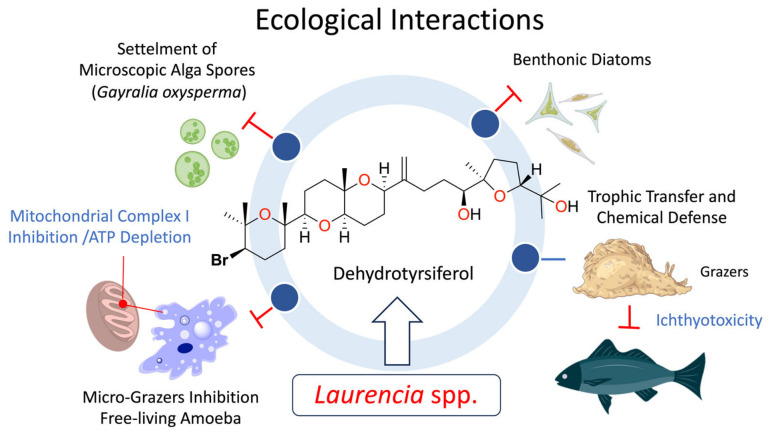
Ecological network and multi-trophic interactions mediated by dehydrothyrsiferol in *Laurencia* spp. DT functions as a versatile, ‘quasi-ready’ chemical weapon deployed across multiple ecological fronts. At the microscopic level, exudation of DT forms a defensive boundary layer that inhibits the settlement of biofouling organisms (green algae and benthic diatoms) and exhibits potent amoebicidal activity via mitochondrial Complex I disruption. Macroscopically, while DT acts as a generalist feeding deterrent, specialist herbivores like the sea hare *Aplysia dactylomela* sequester and metabolically remodel the DT scaffold into potent ichthyotoxins (e.g., aplysiols), effectively re-arming the chemical defense against higher-order predators.

Although in situ ecological measurements of fouling pressure on *L. viridis* are not available, laboratory assays have confirmed that DT exerts potent antifouling activity. Specifically, the compound significantly inhibits the settlement of zoospores of the green alga *Gayralia oxysperma* and the adhesion of benthic diatoms [26]. This specific bioactivity, coupled with the high concentration of the metabolite in *L. viridis*, suggests that DT functions analogously to the chemical shield species described for other *Laurencia* [5], creating a protective boundary against epibionts (Figure 5).

### 5.2. Chemical Defense and Trophic Interactions

General ecological models posit that halogenated terpenoids function as broad-spectrum feeding deterrents against generalist herbivores, while remaining susceptible to sequestration by specialist opisthobranchs [69,70,71,72]. However, the interaction between *L. viridis* and the specialist sea hare *A. dactylomela* reveals a complex co-evolutionary landscape of selective bioaccumulation and metabolic processing.

Evidence from South China Sea populations reveals that *A. dactylomela* accumulates rearranged polyethers, specifically aplysiols A and B, as major metabolites in its mantle, while the typical oxasqualenoids of *Laurencia*, dehydrothyrsiferol and venustatriol, are detected only in trace amounts [66]. This quantitative discrepancy suggests two plausible ecological mechanisms. First, it may support the hypothesis of rapid digestive biotransformation, where the herbivore utilizes the abundant algal metabolite (DT) as a high-value scaffold to generate specialized defensive agents like aplysiols and aplysqualenols [66,67]. Rather than neutralizing the compound, this metabolic remodeling effectively ‘sharpens’ the chemical shield, yielding derivatives with potent ichthyotoxicity and feeding deterrence against higher-order predators [66].

Alternatively, this profile may reflect biogeographic variation in algal metabolism [14]. Given that Laurencia species exhibit significant chemotypic diversity across oceans, it is possible that populations in the Indo-Pacific or Caribbean have evolved to produce distinct oxasqualenoid congeners (such as those resembling aplysiols or aplysqualenols) in response to local pressures, independent of the *L. viridis* chemotype found in Macaronesia [16,25,55,56,57,58,60,61,62,63,64]. Regardless of the origin, the ecological outcome is the same: the successful integration of the oxasqualenoid scaffold into the mollusk’s defensive arsenal confirms its evolutionary efficacy as a deterrent against predation. Manzo et al. (2007) demonstrated that aplysiols A and B showed significant feeding deterrence against the goldfish *Carassius auratus* (at 50 µg/cm^2^) and high ichthyotoxicity against *Gambusia affinis* (10 ppm) [66].

### 5.3. Spatial Competition and Defense Against Micro-Grazers

Beyond macro-herbivores, *L. viridis* engages in microscopic chemical warfare to secure substrate space and maintain surface hygiene. The algae compete directly with fast-growing epiphytes; specifically, DT-mediated inhibition of *G. oxysperma* settlement and diatom adhesion curtails resource availability, effectively displacing micro-herbivores in the competition for substrate space [26]. This allelopathic activity likely creates a ‘zone of inhibition’ around the thallus, preventing epiphytic overgrowth that would otherwise compromise photosynthetic efficiency.

Furthermore, this chemical shield functions as a sanitary defense against protozoan micro-grazers. DT has demonstrated potent amoebicidal activity against *Acanthamoeba* species, effectively preventing colonization by these ubiquitous heterotrophs [38]. Crucially, this lethality is driven by a profound bioenergetic disruption induced by the metabolite. The observed mitochondrial dysfunction in DT-treated cells is mechanistically corroborated by studies on its structural homolog, thyrsiferol, which acts as a specific inhibitor of mitochondrial Complex I of the electron transport chain [73]. Consequently, these oxasqualenoids suppress cellular respiration and ATP synthesis, confirming that DT protects the alga not merely by taste deterrence, but by triggering a targeted collapse metabolism in eukaryotic grazers and pathogens attempting to breach the algal surface.

## 6. Pharmacological Potential: From Ecological Scaffold to Therapeutic Lead

### 6.1. Precision Targeting of Protein Phosphatase 2A

The pharmacological relevance of DT is deeply linked to its role as a specialized structural template within the oxasqualenoid family; in particular, it serves as a highly selective modulator of serine/threonine phosphatases. In comparative enzymatic assays using purified catalytic subunits, DT acts as a moderate but discriminatory inhibitor of Protein Phosphatase 2A (PP2A) [31]. This selectivity is non-trivial; considering the high sequence homology and structural conservation between the catalytic cores of PP1 and PP2A [74,75], the ability of the oxasqualenoid scaffold to distinguish between these nearly identical pockets suggests an evolutionary refinement of the binding interface, likely dictated by subtle conformational interactions with the distinct loop regions surrounding the active site [31,74,76].

**Figure 6:**
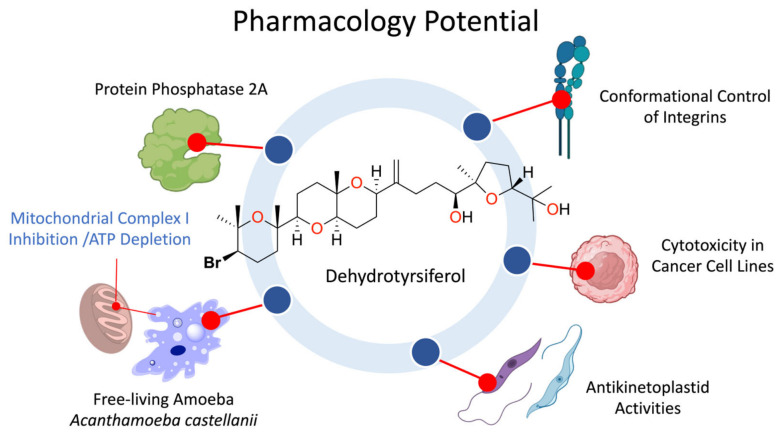
Dehydrothyrsiferol as a multi-target compound.

This structural precision supports the hypothesis of DT acting as a ‘metabolic chassis’ or reservoir. The organism invests substantially in maintaining high tissue concentrations of this moderately active metabolite [29], which remains non-toxic during storage within the CC. However, the functional plasticity of this scaffold is evident in structure-activity relationship (SAR) studies: minor site-specific modifications drastically amplify its inhibitory potency. For instance, hydroxylation at C16, observed in natural congeners like 16-hydroxydehydrothyrsiferol and thyrsenol A, significantly enhances PP2A inhibition [31,76]. This data suggests a biological strategy where the alga maintains a massive, stable chemical reserve (DT) that serves as a near-ready template, capable of being rapidly converted into highly potent toxins through minimal enzymatic tailoring when an acute defensive response is required [31,44,68] (Figure 6). While PP2A modulation has been reported for several *Laurencia*-derived polyethers [52], specific DT docking studies have not been reported.

### 6.2. Conformational Control of Integrins and Induction of Apoptosis

The pharmacological sophistication of DT is perhaps best exemplified by its ability to function as an allosteric modulator of VLA integrins. Unlike non-specific membranolytic agents, DT exerts a defined disruption of cell adhesion dynamics. Experimental evidence in highly invasive breast cancer models (MDA-MB-231) demonstrates that the metabolite markedly impairs adhesion to collagen type I and fibronectin, processes mediated by α2β1 (VLA-2) and α5β1 (VLA-5) integrins, respectively, without altering the surface expression levels of these receptors [32,77]. This suggests a mechanism of conformational freezing, where DT stabilizes the integrin in a low-affinity state, effectively decoupling the cell from the extracellular matrix (ECM).

The specificity of this blockade is absolute. The resulting loss of anchorage triggers Integrin-Mediated Death (IMD), a specialized form of apoptosis characterized by the detachment of cell clusters prior to biochemical execution [32,77]. The causality between integrin conformation and cell death was rigorously confirmed using the monoclonal antibody TS2/16, a biological tool that locks the β1 subunit in a high-affinity state. Remarkably, the external activation of the receptor by TS2/16 completely abolishes DT-induced apoptosis, proving that cell fate is governed directly by the chemical control of the integrin’s conformational switch rather than by off-target toxicity [32,78].

Molecular Docking simulations provide a structural rationale for this activity and further support the biogenetic reservoir hypothesis. The oxasqualenoid scaffold occupies the RGD-binding pocket at the interface of the α/β subunits. However, computational analysis reveals a critical structure-activity nuance: while the natural DT scaffold fits the pocket, derivatives possessing a primary alcohol at C28 (such as 15,28-dihydroxydehydrothyrsiferol) achieve a significantly stronger binding energy by coordinating directly with the divalent cation at the Metal Ion-Dependent Adhesion Site (MIDAS) [36,79]. This observation aligns perfectly with the evolutionary strategy of autotoxicity evasion: the alga stores the moderately active DT (lacking the C28-OH) to prevent lethal interference with its own cellular machinery, yet maintains a scaffold that can be enzymatically oxidized to a highly potent integrin antagonist, capable of blocking the MIDAS site, in response to predation or pathogenic invasion [36,44,68].

### 6.3. Cytotoxicity Profile and Strategic Evasion of Multidrug Resistance (MDR)

The cytotoxic landscape of DT reflects a fascinating paradox of potency and selectivity. While its general antiproliferative profile against solid tumor lines (e.g., A549, HeLa, WiDr) is moderate (GI_50_ 2.3–19 µM) compared to clinical standards [34], this apparent limitation is counterbalanced by an exceptional specificity towards lymphocytic leukemia. In benchmark evaluations against the P-388 murine leukemia model, DT exhibits a strikingly high potency (IC_50_ 0.017 µM) [29], indicating a lineage-selective sensitivity that sets it apart from its broader action on solid tumors.

Structure-activity relationship (SAR) studies have mapped the critical pharmacophores within this scaffold. The integrity of the brominated tetrahydropyran (ring A) and the lipophilicity of the C15–C19 hinge region are non-negotiable; the removal of the bromine atom or the fragmentation of the polycyclic core results in a total loss of bioactivity [34]. Conversely, modifications at C18 (e.g., ketone introduction in 18-ketodehydrothyrsiferol) are well-tolerated, maintaining the cytostatic profile. This SAR data reinforces the view of DT as an ‘evolutionarily optimized lead’: nature has already selected the minimum essential features for target recognition, leaving specific positions (like C16 or C28) available for enzymatic potentiation.

However, the most clinically relevant attribute of the DT scaffold is its ability to bypass Multidrug Resistance (MDR) mechanisms. High-level expression of the P-glycoprotein (P-gp/ABCB1) efflux pump renders many high-potency chemotherapeutics, such as taxanes or anthracyclines, ineffective [80,81]. In stark contrast, functional assays in colchicine-resistant epidermoid carcinoma (KB-8-5) and breast cancer lines (Hs578T) have demonstrated that DT acts as a non-substrate for P-gp [33]. Its antiproliferative efficacy remains remarkably consistent regardless of pump overexpression, indicating that the oxasqualenoid framework effectively ‘evades’ the transporter’s recognition domain. This ‘invisibility’ to cellular defense machinery positions DT as a strategic template for addressing refractory tumors, mirroring the logic of successful non-Pgp substrates like epothilones, and offering a blueprint for designing next-generation agents capable of overcoming chemoresistance [33,82].

### 6.4. Broad-Spectrum Antiprotozoal Activity: Bioenergetic Collapse as a Unifying Mechanism

The versatility of the DT scaffold extends beyond oncology, exhibiting potent activity against a spectrum of protozoan pathogens. Across these divergent eukaryotic lineages, the oxasqualenoid framework appears to target a conserved ‘Achilles’ heel: the mitochondrial bioenergetic machinery.

#### 6.4.1. Amoebicidal Impact and Scaffold Optimization

In models of the opportunistic pathogen *Acanthamoeba castellanii* Neff, DT induces a defined apoptosis-like Programmed Cell Death (PCD). Unlike non-specific biocides that cause necrotic lysis, DT triggers a ‘silent’ metabolic implosion characterized by chromatin condensation, a massive depletion of ATP levels (>80%), and the collapse of the mitochondrial membrane potential (Δψ_m_). Crucially, it exhibits cysticidal activity against the highly resistant cyst stage (IC_50_ 39.27 µM) [38], a clinical rarity as cysts are typically impervious to standard chemotherapy.

The role of DT as a scaffold is most evident in the case of the brain-eating amoeba, *Naegleria fowleri*. While the natural metabolite shows moderate activity, the introduction of a ketone group at C18 (yielding yucatecone) acts as a molecular switch, enhancing potency five-fold. This optimized derivative (IC_50_ 16.25 µM) significantly outperforms the clinical standard miltefosine (IC_50_ 81.57 µM), driving a rapid bioenergetic catastrophe (90% ATP loss) that clears infections where traditional treatments fail [83].

#### 6.4.2. Activity Against Kinetoplastids and In Vivo Validation

The therapeutic potential of DT has been demonstrated against kinetoplastids. In screening against *Trypanosoma cruzi* and *Leishmania amazonensis*, the metabolite emerged as a lead compound, particularly against the clinically relevant intracellular amastigotes of *L*. *amazonensis* (IC_50_ 8.36 µM), comparable to the reference drug miltefosine (IC_50_ 6.48 µM). The toxicity observed for DT against murine macrophage J774.A1 was CC_50_ 28.77 µM. Atomic force microscopy (AFM) analysis of DT-treated *T*. *cruzi* epimastigotes reveals dramatic ultrastructural damage, including loss of intracellular content, flagellar pocket collapse, and extensive membrane blebbing. These morphological effects are consistent with the confirmed mitochondrial membrane potential collapse and profound ATP depletion observed in both *T*. *cruzi* and *L*. *amazonensis* upon DT treatment [37].

Ultrastructural analysis by Transmission Electron Microscopy (TEM) reveals that DT specifically disrupts the acidocalcisomes and causes mitochondrial swelling, corroborating the blockade of respiratory complexes described in other models. Most importantly, DT has successfully bridged the gap from in vitro promise to preclinical efficacy. In a murine model of cutaneous leishmaniasis (*BALB/c*), a topical formulation of DT (0.5% *w*/*v*) achieved an 87% reduction in lesion size after two weeks of treatment, with lesion size evaluated three weeks after treatment completion, with an animal survival rate of 100%. Beyond local healing, the treatment drastically reduced parasite load in the liver and spleen, confirming that the molecule effectively prevents the visceralization of the infection [84]. Although this is currently the only in vivo study reported for DT and further studies are required, the observed high efficacy, selective mitochondrial toxicity, and low systemic absorption support the oxasqualenoid scaffold as a promising candidate for neglected tropical diseases.

## 7. Intellectual Property

A systematic search of the Lens.org, Espacenet, and USPTO databases, using the terms ‘dehydrothyrsiferol’, ‘*Laurencia viridis*’, and ‘triterpene polyethers’, yielded no patent records specifically claiming DT as a protected invention, either as a chemical entity or as an active ingredient in a formulation. To date, no patent has been filed on DT itself, its biological activities, or its synthetic access. This absence of intellectual property protection is not, however, indicative of a lack of scientific or commercial interest; rather, it reflects three compounding realities that define the current stage of DT’s translational trajectory.

First, the structural complexity of DT, encompassing 30 carbons, four fused oxygenated rings, and 9 stereogenic centers, renders its total chemical synthesis an extraordinary challenge that, despite significant efforts [43,59], has not yet yielded a scalable, cost-effective synthetic route. Without reproducible access to multi-gram quantities of pure compounds, the downstream toxicological and pharmacokinetic studies required to support a patent filing with robust efficacy data remain unfeasible.

Second, the natural source, *L. viridis*, is an annual endemic macroalga of Macaronesia with highly seasonal biomass production, restricted geographic range, and sensitivity to environmental stressors [21,22,23]. This constraint has historically limited the biological supply of DT to quantities sufficient for academic research, but inadequate for the sustained preclinical pipelines that precede patent prosecution.

Third, the existing pharmacological evidence, while compelling, including PP2A inhibition, circumvention of P-glycoprotein-mediated multidrug resistance, and an 87% reduction of cutaneous leishmaniasis lesions in murine models [31,33,84], derives predominantly from proof-of-concept studies conducted at a single institution. The breadth of independent replication and comprehensive preclinical validation required to justify the significant financial investment of a patent application has not yet been achieved.

Taken together, the current absence of patent filings should be interpreted as a strategic opportunity rather than a deficiency. The pharmacological targets validated for DT, PP2A, VLA integrins, and mitochondrial electron transport in kinetoplastids are all clinically recognized targets for which alternative inhibitors exist or are under active development. This validates the biological rationale for DT-inspired drug discovery and defines a clear pathway for translational investment. Furthermore, the lack of prior art implies a completely uncrowded intellectual property landscape for this chemical space.

Future patent strategies for DT could reasonably pursue protection of (i) semi-synthetic derivatives with improved synthetic accessibility and retained bioactivity; (ii) formulation technologies enabling stable delivery of lipophilic polyether scaffolds; (iii) biotechnological production platforms, including heterologous expression of the squalene polyepoxidase machinery in microbial hosts; and (iv) specific therapeutic indications, most imminently cutaneous leishmaniasis and drug-resistant hematological malignancies. The current intellectual property landscape thus positions DT not as a compound that has been overlooked, but as one that awaits the synthetic and biotechnological breakthroughs that will unlock its translational potential.

## 8. Conclusions

Dehydrothyrsiferol (DT) exemplifies the *Smart Secondary Metabolite* concept, earning this status not only for its structural intricacy but for its evolutionary role as a versatile metabolic framework. Chemically, it represents a substantial biosynthetic investment by *L. viridis*, whose machinery must drive epoxide-opening cascades from a proposed (6*S*,7*S*,10*R*,11*R*,14*R*,15*R*,18*S*,19*S*)-tetraepoxysqualene precursor to yield a halogenated polyether with a distinctive hook-shaped conformation governed by a critical, flexible C15–C18 bridge that confers the adaptability needed to engage diverse biological targets. Ecologically, DT functions as far more than a simple toxin, acting as a dual-purpose shield that asserts spatial dominance via antifouling activity while serving as a latent chemical weapon against predators, validating the oxasqualenoid core as an evolutionary premium defense scaffold. Pharmacologically, this optimization translates into notable therapeutic promise, with DT behaving as a stealth anticancer agent that evades P-glycoprotein efflux to eliminate multidrug-resistant cells through a multifaceted yet selective mechanism. DT acts as a conformational switch on VLA integrins to trigger apoptosis and as a selective modulator of protein phosphatase 2A (PP2A). DT extends its conserved toxicity to eukaryotic pathogens from free-living amoebae to kinetoplastids by inducing a targeted mitochondrial bioenergetic collapse, a potential already evidenced by an 87% reduction in leishmanial lesions using topical formulations in murine models. In summary, DT is not merely a lead compound but a validated biogenetic template. Although supply remains the principal bottleneck, a clear roadmap positions DT to transition from a marine curiosity to a therapeutic reality through sustained, interdisciplinary efforts that respect its ecological origins while leveraging its pharmacological plasticity.

## Data Availability

No new data were created or analyzed in this study. Data sharing is not applicable to this article.

## Supplementary Materials

The following supporting information can be downloaded at https://www.mdpi.com/article/10.3390/md24050155/s1. Figure S1: Flow diagram of study selection following PRISMA 2020 guidelines; Figure S2: Chemical structures of the oxasqualenoids cited in the manuscript.
