# Supplementary material for: Smart Secondary Metabolites in Marine Environments: Exploring the Oxasqualenoid Dehydrothyrsiferol
Source: Mar Drugs. 2026 Apr 27;24(5):155. doi: 10.3390/md24050155 (PMC13208329; doi:10.3390/md24050155)
Supplement: Supplementary file 1 [file marinedrugs-24-00155-s001.zip › marinedrugs-4234382-supplementary.pdf]

Supplementary Materials

Smart Secondary Metabolites in Marine Environments: Exploring the Oxasqualenoid Dehydrothyrsiferol

Francisco Cen-Pacheco[1,*], Ana R. Díaz-Marrero[2,4*] and José J. Fernández [3,4,5,*]

[1] Facultad de Bioanálisis, Campus-Veracruz, Universidad Veracruzana, Veracruz 91700, Mexico; fcen@uv.mx
[2] Instituto de Productos Naturales y Agrobiología (IPNA), Consejo Superior de Investigaciones Científicas (CSIC), Avenida Astrofísico Francisco Sánchez 3, 38206 La Laguna, Spain; adiazmar@ipna.csic.es
[3] Instituto Universitario de Bio-Orgánica Antonio González (IUBO AG), Universidad de La Laguna (ULL), Avenida Astrofísico Francisco Sánchez 2, 38206 La Laguna, Spain; jjfercas@ull.edu.es
[4] Biotecnología Marina, IUBO-ULL, Unidad Asociada al IPNA-CSIC, 38206 La Laguna, Spain
[5] Departamento de Química Orgánica, Universidad de La Laguna (ULL), Avenida Astrofísico Francisco Sánchez 3, 38206 La Laguna, Spain
* Correspondence: fcen@uv.mx (F.C.P.); adiazmar@ipna.csic.es (A.R.D.M.); jjfercas@ull.edu.es (J.J.F.)

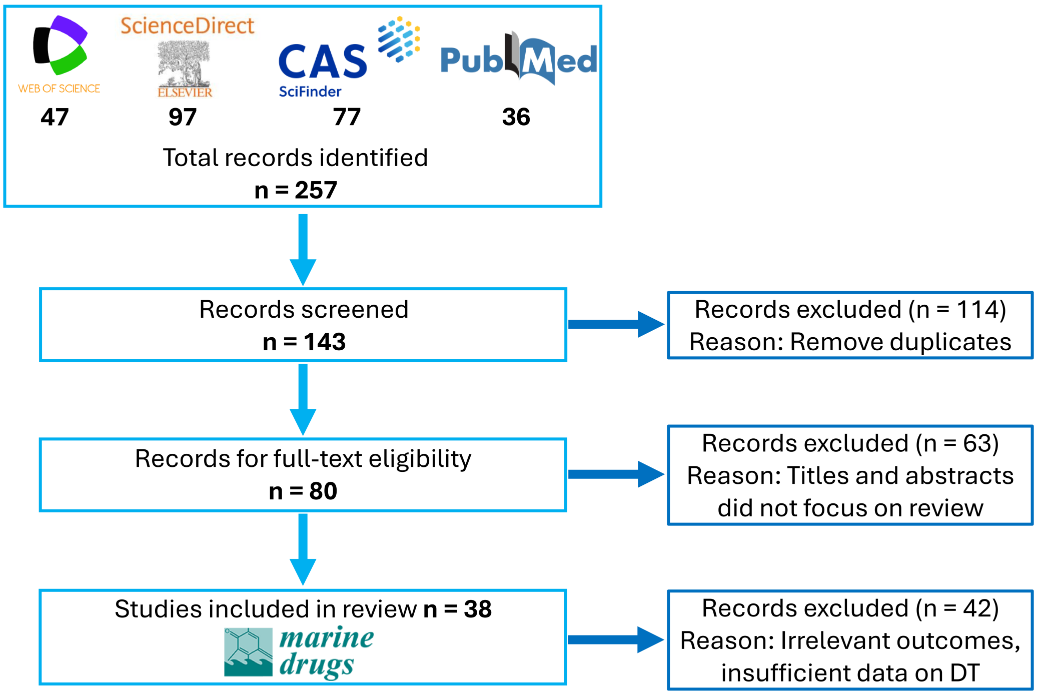

Figure S1. Flow diagram of study selection following PRISMA 2020 guidelines. Records were identified through database searches, duplicates were removed, titles and abstracts were screened, full texts were assessed for eligibility based on predefined criteria, and only studies directly addressing the naturally occurring compound dehydrothyrsiferol were included.

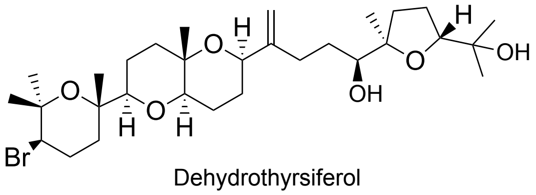

Dehydrothyrsiferol

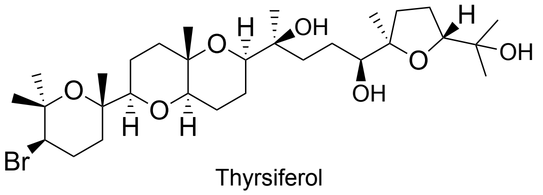

Thyrsiferol

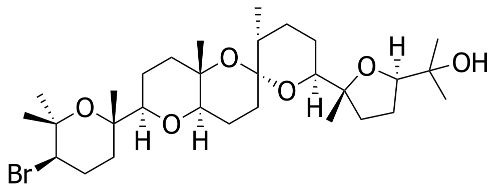

Laurokanol A

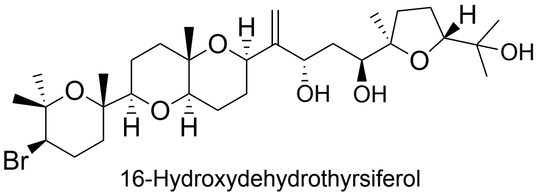

16-Hydroxydehydrothyrsiferol

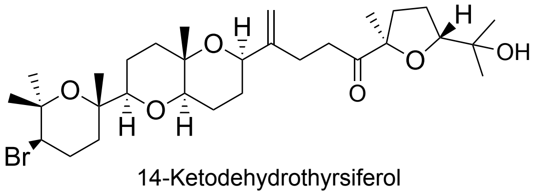

14-Ketodehydrothyrsiferol

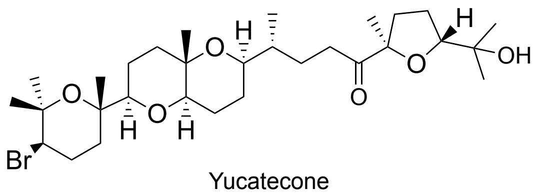

Yucatecone

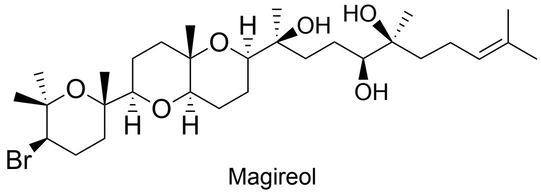

Magireol

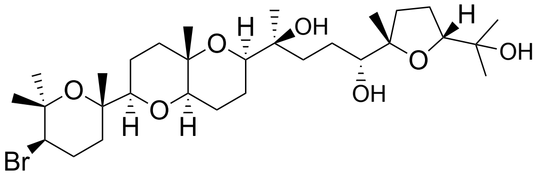

Venustatriol

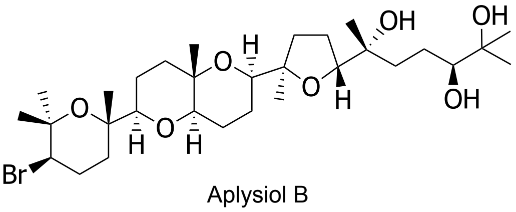

Aplysiol B

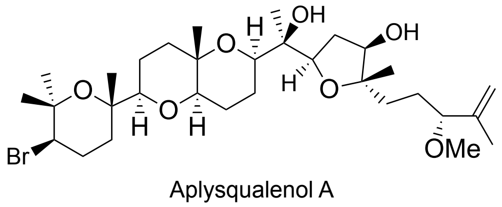

Aplysqualenol A

Figure S2. Chemical structures of the oxasqualenoids cited in the manuscript.

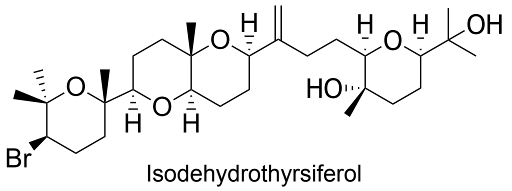

Isodehydrothyrsiferol

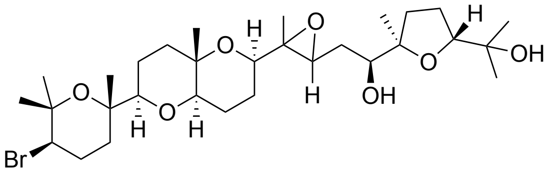

Epoxythyrsiferol A; 15S*, 16R*
Epoxythyrsiferol B; 15R*, 16S*

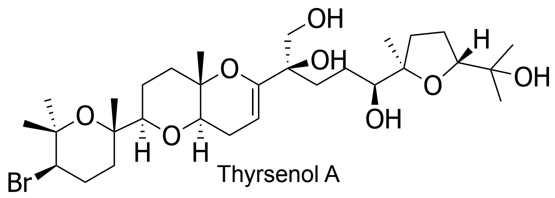

Thyrsenol A

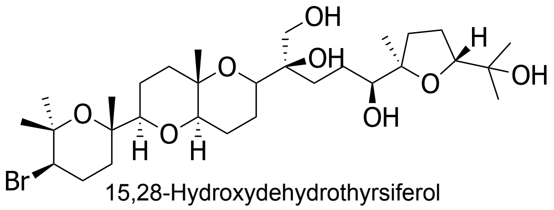

15,28-Hydroxydehydrothyrsiferol

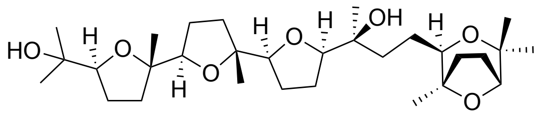

Nivariol

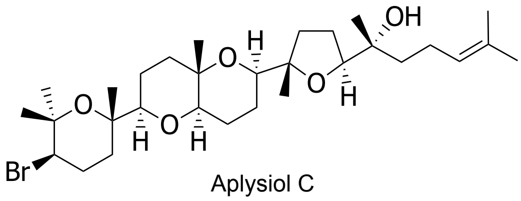

Aplysiol C

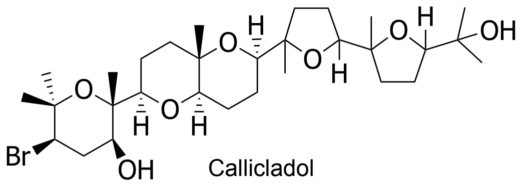

Callicladol

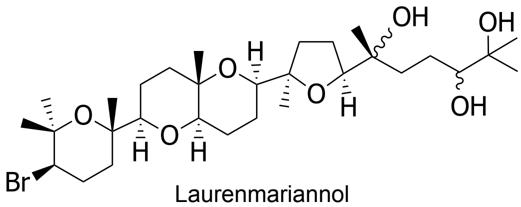

Laurenmariannol

Figure S2 *(cont).* Chemical structures of the oxasqualenoids cited in the manuscript.
